# Evaluation of a prostate SBRT planning workflow using auto‐segmentation and knowledge‐based planning

**DOI:** 10.1002/acm2.70295

**Published:** 2025-10-07

**Authors:** Trisha Jones, Kirk Luca, Mingdong Fan, Pretesh R. Patel, Ashesh B. Jani, Xiaofeng Yang, Justin Roper, Jie Ding

**Affiliations:** ^1^ Department of Radiation Oncology Emory University Atlanta Georgia USA; ^2^ Medical Dosimetry Program School of Health Sciences Southern Illinois University Carbondale Illinois USA

**Keywords:** AI contouring, knowledge‐based planning, Prostate SBRT

## Abstract

**Purpose:**

This study investigates a prostate stereotactic body radiotherapy (SBRT) planning workflow integrating commercial artificial intelligence (AI) contouring and knowledge‐based planning (KBP). The purpose is to determine whether AI‐generated contours are comparable to physician‐delineated contours in achieving dosimetric goals.

**Methods:**

In this retrospective study, 20 patients with intact prostate cancer were included. A commercial AI contouring software was applied to computed tomography (CT) scans. Contouring accuracy for the prostate, rectum, and bladder was assessed against clinical contours using geometric metrics including Dice similarity coefficient (DSC), surface DSC (sDSC), and added path length (APL).

Volumetric modulated arc therapy plans were generated using an in‐house prostate SBRT KBP model without user interaction. The prescribed dose was 36.25 Gy in 5 fractions to the planning target volume with a 40 Gy simultaneous integrated boost to the prostate. All plans were calculated using AcurosXB and normalized to cover 98% of the prostate. Given the high precision required for prostate SBRT, all plans used the physician‐delineated (clinical) prostate contours. Three plans were generated for each patient: (1) a reference plan using clinical contours, (2) a plan using clinical prostate contour and AI organs at risk (OARs), and (3) a plan using clinical prostate contour and post‐processed AI OARs that removed any overlap with the clinical prostate contour. The latter two plans were recalculated on clinical contours with fixed monitor units to evaluate the dosimetric impact of AI contouring. Plan quality was evaluated using NRG‐GU013 criteria.

**Results:**

The average DSC values were 0.83, 0.86, and 0.94 for prostate, rectum, and bladder, respectively, and the average sDSC values were 0.62, 0.81, and 0.85. AI prostate contours were clinically unacceptable. AI rectum and bladder contours overlapped the clinical prostate contour in 15 and 20 cases, respectively.

All reference plans using clinical contours met NRG criteria. Using AI OARs or post‐processed AI OARs, only one case exceeded rectum V36Gy limits due to over‐contouring, but it became clinically acceptable after recalculation on clinical contours. Plans using post‐processed AI OARs yielded dosimetric results more comparable to reference plans for rectum and bladder sparing.

**Conclusions:**

This study investigates a prostate SBRT treatment planning workflow by leveraging AI contouring and KBP. Although a fully automated workflow is not yet feasible, results are encouraging when physician‐delineated target volumes are combined with post‐processed AI contours.

## 1 INTRODUCTION

Prostate cancer is the most common malignancy among men, with an estimated 299,010 new cases in the United States in 2024.^1^ Although incidence rates have been increasing by approximately 0.9% annually, survival outcomes remain favorable, with a 5‐year survival rate of 97.5%.^1^ Given the extended life expectancy following diagnosis, long‐term treatment‐related toxicity is an important consideration when selecting therapeutic approaches. External beam radiation therapy (EBRT) is a standard treatment modality due to its noninvasive nature, preservation of bowel, urinary, and sexual functions, and comparable efficacy to radical prostatectomy.^2^, ^3^, ^4^ Among EBRT techniques, stereotactic body radiotherapy (SBRT) has gained traction. It is considered a standard‐of‐care option for patients with localized prostate cancer, due to its ability to deliver ultra‐hypofractionated treatments with high tumor control and similar toxicity.^5^, ^6^, ^7^ Prostate SBRT is typically prescribed to a dose of 35–40 Gy to the prostate in five fractions, which reduces overall treatment duration and healthcare costs.^8^, ^9^ However, the complexity of SBRT planning presents challenges, as it requires precise delivery of a few high‐dose fractions with steep dose gradients to minimize toxicity.^9^, ^10^

Prostate SBRT presents a unique clinical scenario for evaluating contour accuracy and plan quality due to its use of tighter planning target volume (PTV) margins, higher per‐fraction doses requiring steep dose fall‐off, and more stringent organ at risk (OAR) constraints compared to conventional fractionation. These factors increase sensitivity to even small contour deviations, particularly for adjacent structures such as the rectum and bladder. Consequently, the dosimetric impact of contour accuracy may be more pronounced in SBRT, underscoring the importance of rigorous evaluation in this high‐precision treatment context. Deviations in the delineation of the target and OARs can significantly impact dosimetric outcomes, potentially increasing the risk of recurrence or treatment‐related toxicity.^11^ Manual contouring is labor‐intensive and time‐consuming, posing significant challenges for clinics with high patient volumes and staffing shortages. Similarly, balancing the competing objectives of protecting OARs while achieving sufficient target coverage is often a demanding and time‐intensive aspect of treatment planning. This process is highly dependent on the skill and experience of the planner,^12^, ^13^ leading to potential inconsistencies in plan quality. Although automation has the potential to enhance the efficiency and consistency of both contouring and planning, ensuring the accuracy and reliability of any automated tools is paramount due to their direct impact on patient care.

Auto‐contouring solutions based on artificial intelligence (AI), particularly deep learning, have demonstrated significant improvements in delineation accuracy over traditional automated approaches,^14^ and many institutions have begun evaluating existing commercial AI contouring tools in order to incorporate them into clinical workflows. Geometric metrics such as Dice similarity coefficient (DSC) and Hausdorff distance (HD) are the most popular choices for contour accuracy evaluation; however, they may not strongly correlate with the clinical acceptability of the contours^15^ or the dosimetric endpoints.^16^ Although previous studies have included dosimetric assessments in addition to geometric evaluations to investigate the dosimetric impact of AI contouring tools for prostate treatment planning,^16^, ^17^, ^18^, ^19^, ^20^, ^21^, ^22^, ^23^, ^24^, ^25^ there is a lack of studies focusing specifically on prostate SBRT.

Knowledge‐based planning (KBP) offers a data‐driven planning solution to enhance efficiency and promote consistency across treatment plans.^26^ As an automated approach, KBP leverages predictive dose‐volume histogram (DVH) models developed from curated libraries of high‐quality plans. These models are trained through a systematic process that establishes a mathematical framework to estimate achievable dose distributions for new patients with anatomies similar to those in the training set.^27^The accuracy and utility of KBP depend largely on both the quality of the plans used to build the model and the anatomical similarity between the new patient and the model population.^28^, ^29^ Studies have shown that KBP can consistently generate high‐quality plans, facilitate standardization, and significantly reduce the time and effort required for planning.^29^, ^30^, ^31^, ^32^ Additionally, when combined with auto‐contouring technologies, KBP has the potential to further streamline a fully automated treatment planning workflow that can support consistently high plan quality across clinical settings.^20^

In this study, we investigated a prostate SBRT treatment planning workflow that integrates Radformation AutoContour (Radformation, USA) and RapidPlan KBP (Varian Medical Systems, USA). The purpose of this work is to determine whether AI‐generated contours are comparable to physician‐delineated contours in achieving dosimetric goals.  To the best of the authors’ knowledge, this is the first study to evaluate the dosimetric impact of AI‐generated contours in combination with KBP for prostate SBRT.

## 2 METHODS

### 2.1 Patient selection

Institutional review board approval was obtained for this retrospective study. A total of 20 patients were randomly selected from a cohort of previously treated intact prostate cancer cases between 2013 and 2017. Patients with hip replacements and prostate volumes exceeding 100 cm^3^ (cc) were excluded. Computed tomography (CT) images for 15 patients were acquired using a Siemens Somatom Definition AS CT scanner (Siemens Healthineers, Germany), and images for the remaining patients were acquired using a GE LightSpeed RT 16 CT scanner (GE Healthcare, USA), with the following parameters: matrix size = 512×512, pixel size = 0.86×0.86 ∼ 1.56×1.56 mm^2^, and slice thickness = 2 ∼ 2.5 mm.

The physician‐delineated contours were initially either delineated or reviewed and approved by a radiation oncologist for use in clinical treatment plans and will be referred to as clinical contours in this work. The PTV was created by expanding the prostate clinical target volume (CTV) by 5 mm in all directions, except posteriorly, where a 3 mm expansion was applied. Critical OARs, specifically the rectum and bladder, were included in the analysis for this study.

### 2.2 AI contouring

Auto‐contouring of the prostate, rectum, and bladder was performed on the CT images using Radformation AutoContour software (Version 2.5.14), which is an AI‐driven, deep learning‐based auto‐contouring tool. Overlaps between AI‐generated OARs (AI OARs) and clinical prostate contours were observed in 15 out of 20 cases for the rectum, and in all 20 cases for the bladder. To address this overlapping issue, a post‐processing step was implemented on AI OARs by cropping the overlapping portions with the clinical prostate contour in the Eclipse treatment planning system (TPS) Version 16.1 (Varian Medical Systems, USA). Due to the insufficient accuracy of the AI‐generated prostate contours (see Results), only clinical prostate contours were used for treatment planning. The AI OARs and post‐processed AI OARs were utilized in the subsequent planning steps to evaluate their dosimetric impact.

The accuracy of AI contouring was evaluated against the clinical contours using five geometric metrics: DSC, surface DSC (sDSC),^33^ mean distance to agreement (MDA), 95th percentile HD (HD95), and added path length (APL).^34^ This combination of complementary metrics, both volume‐based and boundary‐based, was chosen to capture different aspects of contour quality across different organs. Among these metrics, sDSC and APL are relatively new and have demonstrated greater clinical relevance due to their stronger correlations with contour editing time compared to other traditional metrics.^33^, ^34^, ^35^ The sDSC measures the surface overlap between the two contours, while the APL calculates the surface length of the clinical contour, which is not captured by the AI contour. A tolerance of 2 pixels was applied in the calculation of sDSC and APL.

### 2.3 Knowledge‐based Planning

All treatment plans were created by using the volumetric modulated arc therapy (VMAT) technique in the Eclipse TPS. The prescribed dose was 36.25 Gy to the PTV with a simultaneous integrated boost (SIB) of 40 Gy to the prostate CTV, delivered in five fractions. Clinical goals were based on the NRG‐GU013 protocol (NCT05946213). Table 1 summarizes the target coverage and dose constraints for the rectum and bladder.

**TABLE 1:** Clinical goals following the NRG‐GU013 protocol, including target coverage and dose constraints for the rectum and bladder.

Structure	Dosimetric parameter	Per protocol	Variation acceptable
Prostate CTV	V40Gy	98%	–
PTV	V36.25 Gy	≥ 98%	–
Rectum	V36Gy	≤ 1cc	< 3cc
	V32.6 Gy	≤ 10%	≤ 15%
	V29Gy	≤ 20%	< 25%
	V18.1 Gy	≤ 50%	< 55%
Bladder	V37Gy	≤ 10cc	< 20cc
	V18.1 Gy	≤ 40%	< 45%

Abbreviations: CTV, clinical target volume; PTV, Planning target volume.

A KBP RapidPlan model was developed in‐house for prostate SBRT using 96 high‐quality plans following the NRG‐GU013 protocol. None of the cases included in this study were used in the development or training of the KBP model. Tables S1‐S3 provide the KBP model's custom optimization template for target coverage and OAR dose objectives, as well as fine‐tuned normal tissue objectives (NTOs) to optimize the dose gradient outside of the target. For each patient in this study, three plans were automatically generated using this established in‐house RapidPlan model with no user interaction beyond structure matching: (1) a reference plan with clinical contours, (2) a plan with clinical prostate CTV and AI OARs, and (3) a plan with clinical prostate CTV and post‐processed AI OARs. All plans were created using three coplanar arcs with collimator angles of 350°, 10°, and 90°, employing 10 MV flattening‐filter‐free (FFF) beams with a dose rate of 2400 MU/min on a Varian TrueBeam linear accelerator. A maximum monitor unit (MU) objective of 2400, with a priority of 50, was applied prior to optimization. RapidPlan optimization was performed with convergence mode set to “ON”. Dose calculations were performed using the AcurosXB algorithm for both intermediate and final dose calculations, with a dose grid resolution of 1 mm. Following the final dose calculation, plan normalization was applied to ensure that 98% of the prostate CTV received 40 Gy.

Plans using AI OARs and post‐processed AI OARs were also recalculated on the clinical contours with fixed MUs to evaluate the dosimetric impact of AI contouring. Plan quality was assessed by clinical goals and also compared to the reference plan with clinical contours, focusing on the target coverage and dose constraints for the rectum and bladder. A paired *t*‐test was performed to determine whether the differences in dosimetric endpoints relative to the reference plan were statistically significant (*p* < 0.05 considered significant).

## 3 RESULTS

### 3.1 AI contouring evaluation

**FIGURE 1:**
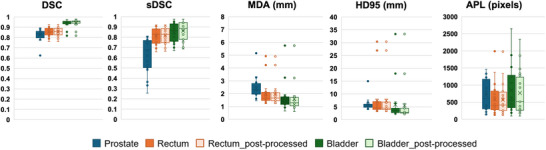
Boxplots of the geometric metrics for the prostate, rectum and bladder.

Figure 1 presents the five geometric evaluations (DSC, sDSC, MDA, HD95, and APL) for the prostate, rectum, and bladder AI contours compared to the clinical contours. Table 2 summarizes the mean values and standard deviations of these geometric metrics across all 20 patients. Minimal improvements in the geometric metrics were observed for the post‐processed AI OARs.

**TABLE 2:** Geometric metrics for the prostate, rectum, and bladder.

		AI OARs		Post‐processed AI OARs	
Mean ± (std)	Prostate	Rectum	Bladder	Rectum	Bladder
DSC	0.83 (± 0.06)	0.86 (± 0.04)	0.94 (± 0.04)	0.86 (± 0.04)	0.94 (± 0.04)
sDSC	0.62 (± 0.17)	0.81 (± 0.08)	0.85 (± 0.09)	0.82 (± 0.08)	0.86 (± 0.09)
MDA (mm)	2.42 (± 0.82)	1.99 (± 0.95)	1.64 (± 1.10)	1.98 (± 0.94)	1.59 (± 1.11)
HD95 (mm)	5.87 (± 2.35)	7.44 (± 7.42)	5.82 (± 7.33)	7.34 (± 7.45)	5.52 (± 7.38)
APL (pixels)	699 (± 449)	577(± 468)	850 (± 671)	568 (± 463)	769 (± 625)

Abbreviations: APL, added path length; DSC, Dice similarity coefficient; HD, Hausdorff distance; MDA, mean distance to agreement; sDSC, surface DSC.

**FIGURE 2:**
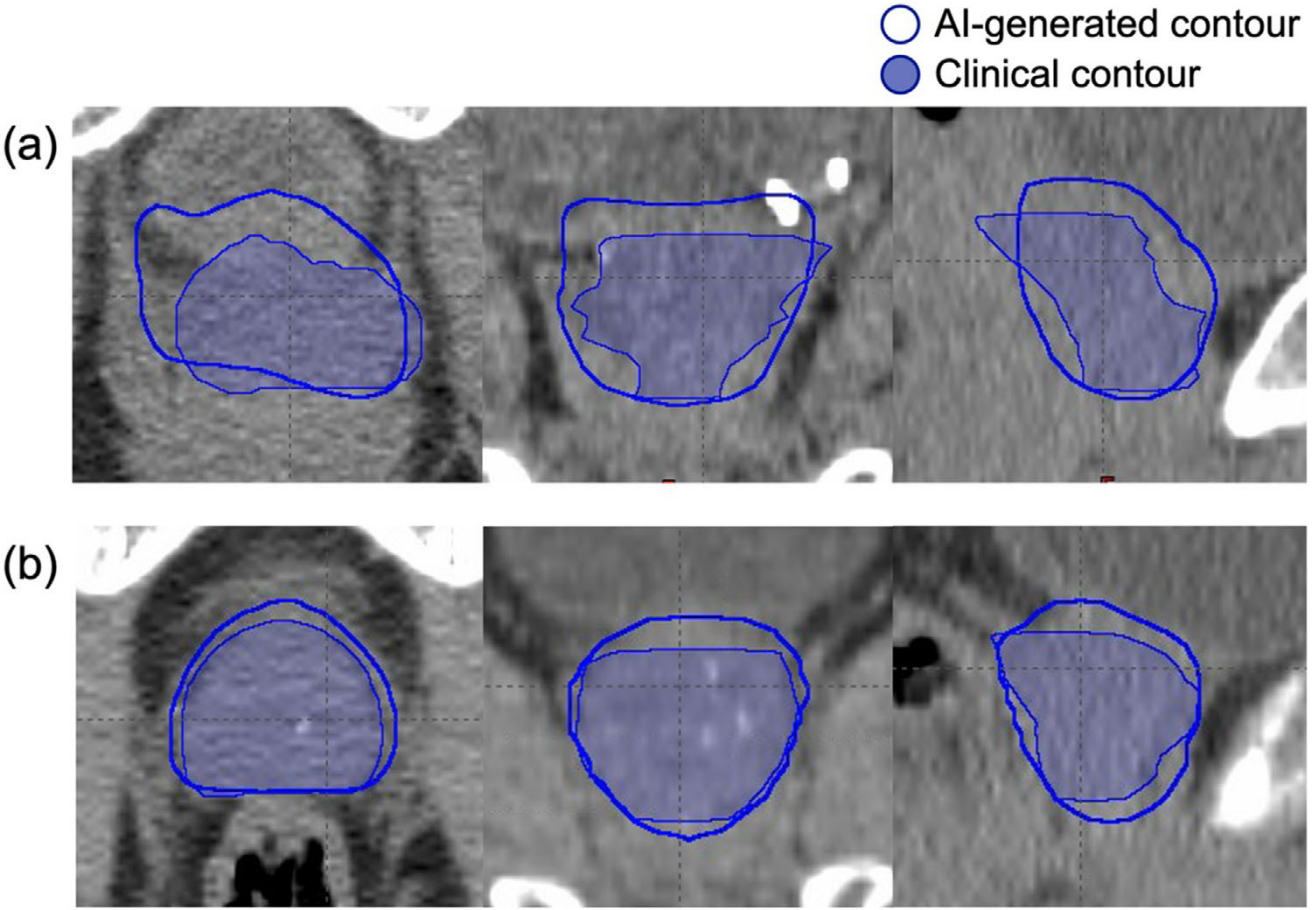
Example cases of AI‐generated prostate contours compared to the clinical prostate contours (filled) in three views: (a) case with the worst sDSC (0.26), and (b) case with the best sDSC (0.81). AI, artificial intelligence; sDSC, surface DSC.

AI‐generated prostate contours demonstrated unsatisfactory accuracy, particularly with an average sDSC of only 0.62. Figure 2 illustrates two example cases: one with the worst sDSC and one with the highest sDSC. Even in the case with the best sDSC, over‐contouring of the prostate was observed. Given the high fractional dose in prostate SBRT, the AI‐generated prostate contours were not suitable for direct use in treatment planning.

**FIGURE 3:**
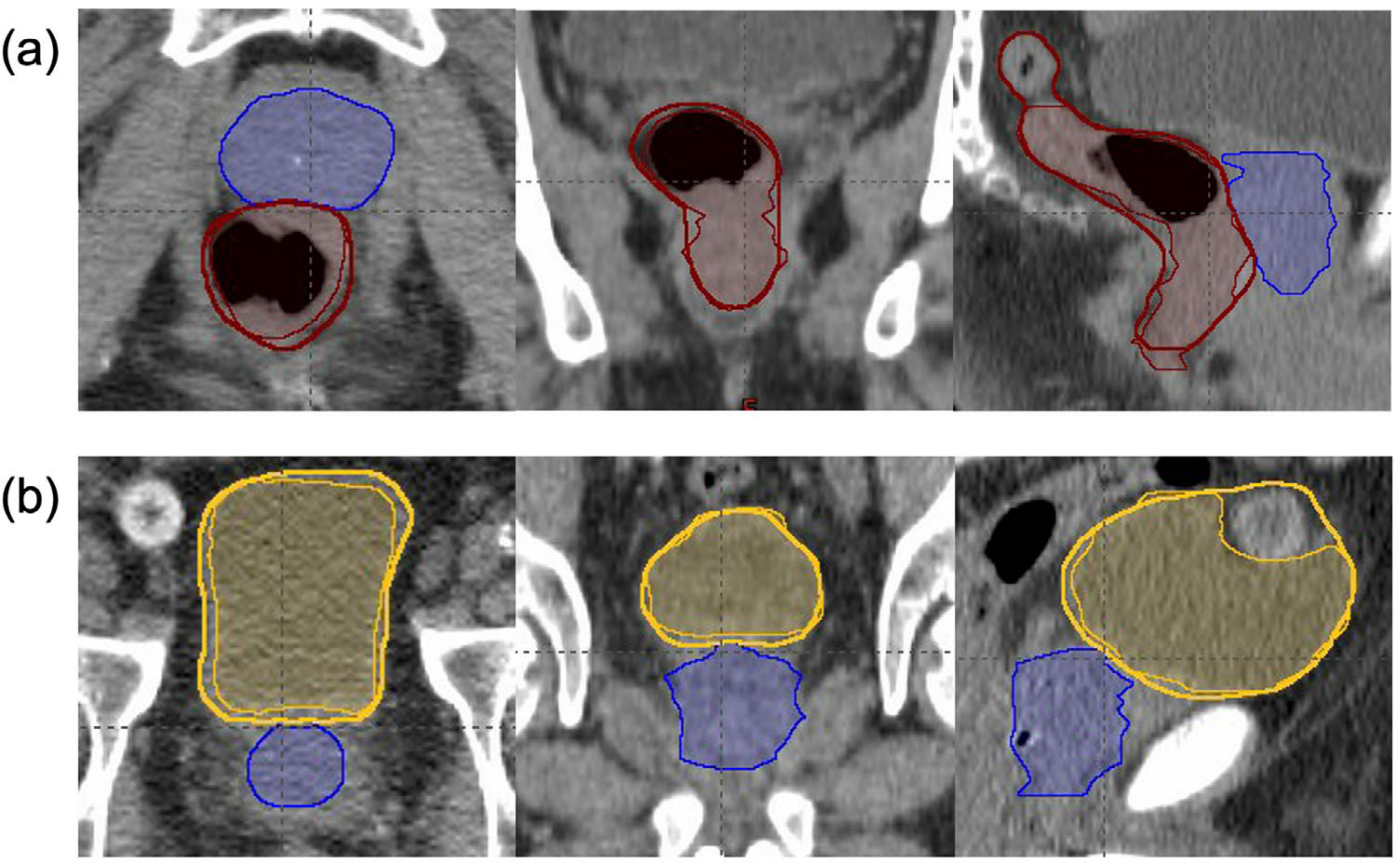
Example cases of AI‐generated OARs in three views: (a) rectum (in red) compared to the clinical contour (filled), and (b) bladder (in yellow), compared to the clinical contour (filled). The clinical prostate contours (in blue) are also displayed. AI, artificial intelligence; OARs, organs at risk.

AI‐generated OARs achieved mean DSC > 0.85 and sDSC > 0.80 for both the rectum and bladder. The suboptimal performance of AI OARs was primarily due to over‐contouring, as shown in the examples in Figure 3. This over‐contouring also resulted in overlaps with clinical prostate contours, which occurred in 15 out of 20 cases for the rectum and all 20 cases for the bladder. For the rectum, discrepancies in the most superior slice compared to the clinical contour led to two outliers in MDA and HD95, as shown in the example in Figure 3a. Similarly, for the bladder, the inclusion of adjacent structures caused the outliers in MDA and HD95, as shown in the example in Figure 3b (the AI bladder contour includes the reservoir of a penile implant).

### 3.2 Dosimetric evaluation of RapidPlan‐generated plans

#### 3.2.1 Target coverage

**FIGURE 4:**
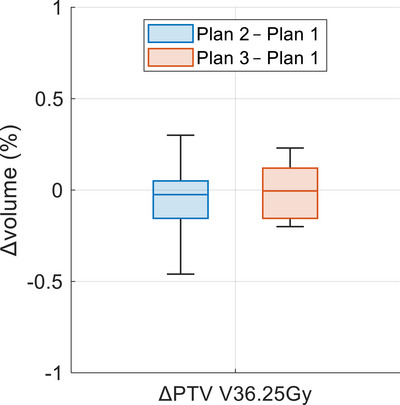
The difference in PTV V36.25 Gy (%) relative to the reference plan. Plan 1 (reference plan) with clinical contours, Plan 2 with AI OARs, Plan 3 with post‐processed AI OARs. The maximum difference was < 0.5%. AI, artificial intelligence; OARs, organs at risk.

All treatment plans were generated using the clinical prostate CTV and its corresponding PTV, with normalization applied to ensure 98% of the prostate CTV received 40 Gy (V40Gy = 98%). The PTV coverage of V36.25 Gy ≥ 98% was achieved in all plans. Table 3 presents the PTV V36.25 Gy values for all three plans generated by the RapidPlan model, demonstrating comparable results across all 20 cases. Figure 4 illustrates the differences in PTV V36.25 Gy relative to the reference plan, and no statistically significant differences were observed (*p*‐values > 0.05).

**TABLE 3:** PTV V36.25 Gy (%) in the three plans generated by the RapidPlan model.

PTV V36.25 Gy (%)	Mean ± (std)	Range
**Plan 1**–clinical contours (reference plan)	98.65 (± 0.24)	[98.22, 99.05]
**Plan 2**–clinical prostate CTV, AI OARs	98.62 (± 0.32)	[98.01, 99.24]
**Plan 3**–clinical prostate CTV, post‐processed AI OARs	98.64 (± 0.30)	[98.02, 99.12]

Abbreviations: AI, artificial intelligence; CTV, clinical target volume; OARs, organs at risk; PTV, Planning target volume.

#### 3.2.2 Rectum dose constraints

For the rectum dose constraint of V36Gy ≤ 1 cc (with < 3 cc considered variation acceptable), the reference plan met the constraint in 12 patients, with the remaining 8 patients classified as variation acceptable. The plan using AI OARs met the goal in 9 patients, achieved variation acceptable in 10 patients and failed in 1 patient. The plan using post‐processed AI OARs met the goal in 11 patients, achieved variation acceptable in 8 patients and failed in 1 patient. After recalculating the plans with AI OARs and post‐processed AI OARs on the clinical contours, both recalculated plans met the goal in 10 patients, with the remaining 10 patients showing variation acceptable and no failures. All other rectum dose constraints –V32.6 Gy ≤ 10%, V29Gy ≤ 20%, and V18.1 Gy ≤ 50%–were met in all three plans generated by the RapidPlan model (reference plan with clinical contours, plan with AI OARs, and plan with post‐processed AI OARs), as well as in the recalculated plans.

**FIGURE 5:**
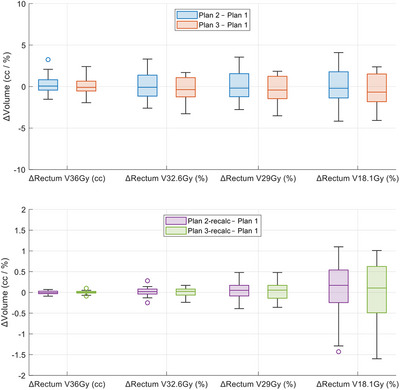
Differences in rectum dosimetric endpoints relative to the reference plan. Plan 1 (reference plan) with clinical contours, Plan 2 with AI OARs, Plan 3 with post‐processed AI OARs. The suffix “‐recalc” indicates the plan was recalculated on clinical contours. AI, artificial intelligence; OARs, organs at risk.

Table 4 summarizes the rectum dosimetric endpoints for all five plans. Figure 5 displays the differences in rectum dosimetric endpoints relative to the reference plan, and no statistically significant differences were observed (all p‐values > 0.05).

**TABLE 4:** Rectum dosimetric endpoints in the three plans generated by the RapidPlan model, along with the recalculated plans using clinical contours. Plan 1 (reference plan) with clinical contours, Plan 2 with AI OARs, Plan 3 with post‐processed AI OARs.

	V36Gy (cc)		V32.6 Gy (%)		V29Gy (%)		V18.1 Gy (%)	
Rectum dosimetric endpoints	Mean (± std)	Range	Mean (± std)	Range	Mean (± std)	Range	Mean (± std)	Range
Plan 1	1.05 (± 0.72)	[0.10, 2.72]	2.42 (± 1.31)	[0.76, 5.64]	3.56 (± 1.57)	[1.60, 7.25]	9.64 (± 2.70)	[5.88, 15.29]
Plan 2	1.32 (± 1.06)	[0.05, 4.77]	2.55 (± 1.27)	[0.65, 5.60]	3.69 (± 1.51)	[1.45, 7.20]	9.62 (± 3.01)	[4.71, 14.47]
Plan 3	1.08 (± 0.86)	[0.05, 3.93]	2.22 (± 1.01)	[0.66, 4.22]	3.33 (± 1.25)	[1.25, 5.69]	9.19 (± 2.85)	[4.74, 14.20]
Plan 2‐recalc ^*^	1.05 (± 0.73)	[0.08, 2.70]	2.43 (± 1.32)	[0.66, 5.67]	3.60 (± 1.60)	[1.42, 7.34]	9.70 (± 2.76)	[5.98, 14.69]
Plan 3‐recalc ^*^	1.06 (± 0.73)	[0.08, 2.72]	2.43 (± 1.31)	[0.67, 5.69]	3.58 (± 1.59)	[1.44, 7.34]	9.63 (± 2.73)	[6.04, 14.49]

* ‐recalc: plan recalculated on clinical contours.

Abbreviations: AI, artificial intelligence; OARs, organs at risk.

The failure of the plans with AI OARs and post‐processed AI OARs to meet rectum V36Gy < 3 cc in one patient was attributed to over‐contouring of the rectum, which resulted in overlap with the clinical prostate CTV. Even after cropping the overlapping portion, the post‐processed rectum remained too close to the clinical prostate CTV, leading to failure of the constraint.

#### 3.2.3 Bladder dose constraints

The bladder dose constraint of V37Gy ≤ 10 cc (with < 20 cc considered variation acceptable) was achieved in the reference plan for 19 patients, with the remaining 1 patient classified as variation acceptable. The plan using AI OARs met the goal in 16 patients, with variation acceptable for the remaining 4. The plan using post‐processed AI OARs met the constraint in 19 patients, with 1 patient showing variation acceptable–matching the performance of the reference plan. No failures occurred in any plans with AI OARs or post‐processed AI OARs. After recalculating the plans, AI OARs and post‐processed AI OAR plans on the clinical contours, both recalculated plans met the goal in 19 patients, and only 1 patient showed variation acceptable–matching the reference plan. The other bladder dose constraint, V18.1 Gy ≤ 40%, was met in all plans, including the recalculated plans.

**FIGURE 6:**
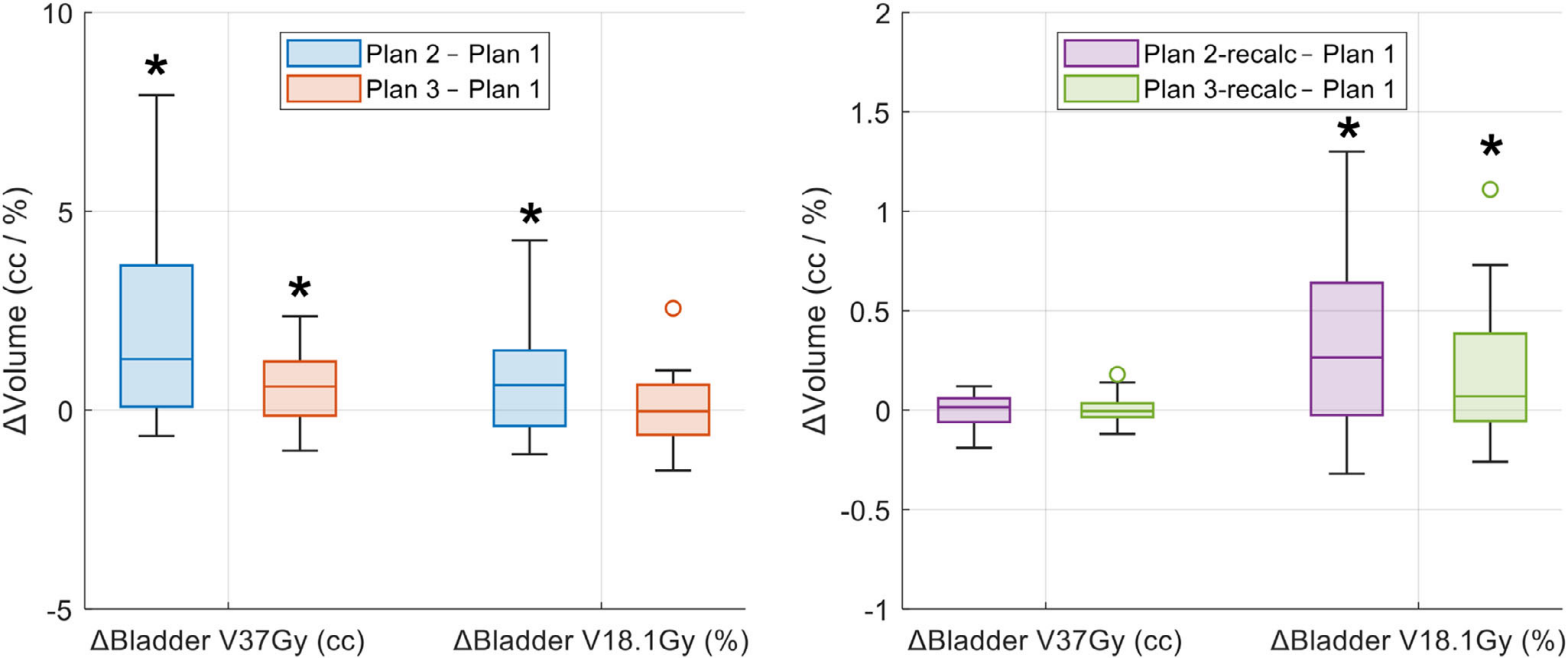
Differences in bladder dosimetric endpoints relative to the reference plan. Significant differences (*p*‐value < 0.05) are marked with asterisks, and the exact p‐values are provided in Table 5. Plan 1 (reference plan) with clinical contours, Plan 2 with AI OARs, Plan 3 with post‐processed AI OARs. The suffix “‐recalc” indicates the plan was recalculated on clinical contours. AI, artificial intelligence; OARs, organs at risk.

Table 5 summarizes the bladder dosimetric endpoints for all five plans, with significant differences (*p* < 0.05) observed when compared to the reference plan. Figure 6 illustrates the differences in bladder dosimetric endpoints relative to the reference plan, with significant differences marked. These results indicate that using AI OARs or post‐processed AI OARs tends to overestimate the dose to the bladder due to over‐contouring. Although significantly higher bladder dose was still observed in V18.1 Gy after recalculating with the clinical contours, the maximum difference was less than 1.5% as shown in Figure 6. Compared to the plan using AI OARs directly, the plan using post‐processed AI OARs achieved bladder dosimetric endpoints more aligned with the reference plan using clinical contours.

**TABLE 5:** Bladder dosimetric endpoints in the three plans generated by the RapidPlan model, along with the recalculated plans on clinical contours. *p*‐values are provided to indicate the significant differences compared to the reference plan. Plan 1 (reference plan) with clinical contours, Plan 2 with AI OARs, Plan 3 with post‐processed AI OARs.

	V37Gy (cc)			V18.1 Gy (%)		
Bladder dosimetric endpoints	Mean (± std)	Range	*p*‐value (vs. Plan 1)	Mean (± std)	Range	*p*‐value (vs. Plan 1)
Plan 1	4.24 (± 2.44)	[0.43, 10.72]	NA	13.46 (± 7.14)	[4.06, 26.09]	NA
Plan 2	6.33 (± 4.01)	[0.89, 14.72]	<0.001	14.26 (± 7.60)	[4.51, 29.74]	0.02
Plan 3	4.84 (± 2.57)	[0.79, 11.00]	0.01	13.50 (± 7.06)	[4.49, 26.94]	>0.05
Plan 2‐recalc^*^	4.24 (± 2.43)	[0.51, 10.62]	>0.05	13.81 (± 7.40)	[3.99, 27.09]	0.002
Plan 3‐recalc^*^	4.25 (± 2.45)	[0.51, 10.71]	>0.05	13.64 (± 7.30)	[4.06, 26.44]	0.03

* ‐recalc: plan recalculated on clinical contours.

Abbreviations: AI, artificial intelligence; OARs, organs at risk.

### 3.3 Ad hoc correlation analysis between geometric and dosimetric performance

An ad hoc analysis was performed to investigate the correlations between geometric metrics and dosimetric endpoints using Pearson correlation (*p* < 0.05 considered significant). Significant correlations were observed in the rectum between geometric metrics, sDSC and APL, and dosimetric endpoints. Although these findings suggest potential predictive value of the geometric metrics in dosimetric outcomes, this analysis was exploratory and based on a limited dataset. The details of these exploratory results are provided in the Figures S2 and S3.

## 4 DISCUSSION

Given the stringent accuracy and clinical effort required for prostate SBRT planning, rigorous evaluation and validation of automation tools are essential before clinical adoption. In this study, AI contouring on CT images for the prostate, rectum, and bladder was evaluated using five geometric metrics. Our results revealed its insufficient accuracy in prostate contouring for clinical use in prostate SBRT planning, as indicated by an average sDSC of 0.62 and the visual assessment shown in Figure 2. For critical OARs, a significant concern was the software's tendency to over‐contour the rectum and bladder, resulting in overlap with the clinical prostate contour in 15 out of 20 cases for the rectum and all 20 cases for the bladder. These suboptimal OAR contours impacted clinical goals in RapidPlan treatment planning, with several cases showing dosimetric endpoints that fell into minor deviations but remained clinically acceptable, and only one case showing a rectum V36Gy in the unacceptable range. This unacceptable rectum V36Gy, however, was due to over‐contouring of the AI‐generated rectum contour, and it became clinically acceptable after recalculating dose on the clinical contours. In addition, using the post‐processed AI‐generated rectum and bladder contours (with overlap from the clinical prostate contour removed), the plans yielded dosimetric results more comparable to reference plans in terms of target coverage and OAR sparing.

In clinical practice, it is common for physicians to contour the prostate on magnetic resonance imaging (MRI), particularly T2‐weighted MRI, due to the superior soft tissue contrast compared to CT, allowing for more accurate delineation of the prostate gland.^36^, ^37^ The MRI scan needs to be registered to the planning CT scan, and the prostate contour is then transferred to the CT and refined as necessary. Radformation has provided AI contouring for the prostate using T2‐weighted MRI. However, based on our preliminary test with the first five patients in our cohort, the AI‐generated prostate contours on MRI were even worse than those obtained from CT, which showed an average DSC of 0.59 and sDSC of 0.30, and one case showed complete surface mismatch with the clinical prostate contour (sDCS = 0). Detailed results of this preliminary test are provided in Table S4 and Figure S1. These results demonstrate that AI contouring on MRI is not yet clinically viable for our use. It should be noted that auto‐segmentation on MRI can be particularly challenging, as its performance is significantly impacted by variability in data acquisition, including differences in imaging parameters, acquisition protocols, and MRI vendors.^38^, ^39^ In addition, the inherent registration limitations between MRI and CT can introduce contouring uncertainties, and MRI may also not be accessible for all patients receiving prostate radiotherapy.^40^ Therefore, in this study, we focused on evaluating AI contouring using CT images. Despite our promising findings on CT images, a fully automated workflow for prostate SBRT is not yet feasible due to the limited accuracy of AI contouring. This is consistent with existing studies on online adaptive prostate SBRT workflows, which have primarily relied on manual contour adaptation.^41^, ^42^, ^43^, ^44^, ^45^, ^46^ Although several studies have employed AI contouring, all have shown that manual editing is still required,^47^, ^48^, ^49^ particularly for the CTV contour.^49^ Further improvements are needed to develop an auto‐contouring tool capable of generating highly precise structures–including target and OARs–without human intervention, which remains a critical requirement for achieving a fully automated planning workflow.

One of the central challenges in prostate SBRT planning is delivering a highly conformal dose to the target while minimizing exposure to surrounding OARs, all while maintaining relatively uniform target coverage. This balance is particularly important due to the anatomical complexity of the prostate and its proximity to critical structures. Unlike lung SBRT, which is typically ablative, prostate SBRT requires precise avoidance of the urethra, which lies centrally within the prostate gland. Excessive dose to the urethra can result in significant genitourinary toxicity, making dose sparing in this region a clinical priority.^50^ In this study, the urethra was not contoured in any of the treatment plans—including the original clinical plans—due to its poor visibility on standard CT imaging. Despite this, our KBP model was trained using 96 high‐quality prostate SBRT plans adhering to the NRG‐GU013 protocol, in which the urethra and an associated planning risk volume (PRV) were present in the structure set (as detailed in Table S3). For cases without a urethra structure, our model's optimization template still assigns balanced objectives and priorities to the target structures and incorporates well‐constructed NTO settings to promote dose homogeneity and minimize hot spots (see Tables S1 and S2). In this study, the model consistently constrained the global maximum dose to below 43.2 Gy within 0.03 cc in all 20 cases, which meets the 43.5 Gy constraint for the urethra specified in NRG‐GU013. This indicates the model's ability to reflect urethra sparing even when the structure is not explicitly delineated. Importantly, the model demonstrated the capability to generate high‐quality, clinically acceptable plans without requiring any manual editing or user interaction. With a single click, plans were produced in under 15 min, each exhibiting excellent dose homogeneity, high conformity, and effective sparing of critical OARs. These findings underscore the potential of well‐trained KBP models to streamline and standardize prostate SBRT planning, even in the absence of complete structure sets, and support the broader goal of advancing towards a fully automated treatment planning workflow.

In this specific study and patient cohort, significant correlations were found between geometric metrics (sDSC and APL) and rectal dosimetric endpoints, suggesting that a smaller sDSC or a higher APL (indicating worse AI contouring performance) could potentially predict increased rectal dose and key dosimetric outcomes. However, it remains unclear whether this relationship was driven by the over‐contouring of the AI‐generated rectum contours, or by the variations in patient anatomy and rectal filling. It is also noted that even though the sDSC and APL are promising and more clinically relevant, they are still limited by their intrinsic inability to distinguish where the variations occur within a contour.^51^ Further development of geometric metrics is needed to spatially localize contour variations, such as region‐weighted or distance‐to‐target‐weighted spatial metrics that better capture the contour discrepancies in clinically relevant areas, particularly those adjacent to or closer to the target for OARs.

Despite the promising findings presented, this study has several limitations. The plan quality evaluation in this work mainly relied on DVH parameters, without assessing the spatial dose distributions. The plans also lacked critical verification steps in clinical practice, such as patient‐specific quality assurance, which are essential to ensure delivery accuracy and high‐quality patient care. In addition, there was an absence of clinical plans for direct comparison. Instead, reference plans were generated using the clinically approved RapidPlan model with physician‐delineated clinical contours. Future work will involve a larger patient cohort and more comprehensive evaluations of clinical acceptability in a real clinical workflow ideally with the inclusion of MRI‐guided contours.

## 5 CONCLUSION

This study investigates a prostate SBRT treatment planning workflow leveraging Radformation AI contouring and RapidPlan KBP in the Eclipse TPS. Although a fully automated workflow is not yet feasible, results are encouraging when physician‐delineated target volumes are combined with post‐processed AI‐generated OAR contours. Only one case exceeded the rectum V36Gy constraint due to over‐contouring, which became clinically acceptable after recalculation on clinical contours. Plans using post‐processed AI OARs demonstrated dosimetric results more comparable to reference plans, particularly in terms of rectum and bladder sparing. The combination of AI contouring and KBP provides a pathway to potentially enhance planning efficiency while maintaining plan quality, marking an important step towards a fully automated planning approach for prostate SBRT.

## AUTHOR CONTRIBUTIONS

**Trisha Jones**: Investigation, Methodology, Writing–Original Draft **Kirk Luca**: Supervision, Conceptualization, Investigation, Methodology, Validation, Writing–Original Draft, Writing–Review & Editing **Mingdong Fan**: Writing–Original Draft, Writing–Review & Editing **Pretesh R. Patel**: Writing–Review & Editing **Ashesh B. Jani**: Writing–Review & Editing **Xiaofeng Yang**: Writing–Review & Editing **Justin Roper**: Methodology, Writing–Review & Editing **Jie Ding**: Supervision, Conceptualization, Investigation, Methodology, Validation, Formal analysis, Writing–Original Draft, Writing–Review & Editing.

## CONFLICT OF INTEREST STATEMENT

The authors declare no conflicts of interest.

## Supporting information



Supporting Information

## Data Availability

Research data are stored in an institutional repository and will be shared upon request to the corresponding author.
