# Supplementary material for: Evaluation of a prostate SBRT planning workflow using auto‐segmentation and knowledge‐based planning
Source: J Appl Clin Med Phys. 2025 Oct 7;26(10):e70295. doi: 10.1002/acm2.70295 (PMC12504046; doi:10.1002/acm2.70295)
Supplement: Supplementary file 1 — Supporting Information [file ACM2-26-e70295-s001.docx]

# **Supplementary Material**

**Table S1.** Summary of dose objectives for target structures for the in-house prostate SBRT RapidPlan model.

| Structure ID | Volume [%] | Dose [%] | Priority |
| --- | --- | --- | --- |
| CTV_4000 |  |  |  |
| Upper | 0 | 103 | 185 |
| Lower | 100 | 102 | 185 |
| PTV_3625 |  |  |  |
| Lower | 100 | 102 | 200 |
| z_PTV_3625* |  |  |  |
| Upper | 0 | 103 | 200 |
| Lower | 100 | 102 | 200 |
| * z_PTV_3625 is defined as PTV_3625 cropped 1 mm inside the CTV_4000 | | | |

**Table S2.** Summary of normal tissue objective settings for the in-house prostate SBRT RapidPlan model.

|  | Priority | 200 |
| --- | --- | --- |
|  | Distance from Target Boarder | 0.00 cm |
|  | Start Dose | 100% |
|  | End Dose | 35% |
|  | Fall-Off | 0.18 mm^-1^ |

**Table S3.** Summary of dose objectives for organs at risk for the in-house prostate SBRT RapidPlan model.

| **Structure ID** | **Volume [%]** | **Dose [%]** | **Priority** | **gEUD a** |
| --- | --- | --- | --- | --- |
| **Bag_Bowel** |  |  |  |  |
| Upper | 0 | Generated | 150 |  |
| Upper gEUD* |  | Generated | 70 | 1 |
| Line |  |  | 100 |  |
| **Bladder** |  |  |  |  |
| Upper | 0 | 103 | 350 |  |
| Upper | 0.1 | 102.9 | 150 |  |
| Upper | 10 | 45 | 110 |  |
| Upper gEUD |  | Generated | 80 | 1 |
| Line |  |  | 110 |  |
| **Fem_Head_L** |  |  |  |  |
| Upper gEUD |  | Generated | 80 | 4 |
| Upper gEUD |  | Generated | 80 | 8 |
| Line |  |  | 100 |  |
| **Fem_Head_R** |  |  |  |  |
| Upper gEUD |  | Generated | 80 | 4 |
| Upper gEUD |  | Generated | 80 | 8 |
| Line |  |  | 100 |  |
| **PenileBulb** |  |  |  |  |
| Upper gEUD |  | Generated | 50 | 1 |
| Line |  |  | 70 |  |
| **Rectum** |  |  |  |  |
| Upper | 0 | 103 | 350 |  |
| Upper | 0.1 | 102.9 | 150 |  |
| Upper gEUD |  | Generated | 100 | 1 |
| Line |  |  | 120 |  |
| **Urethra** |  |  |  |  |
| Upper | 0 | 103 | 350 |  |
| **Urethra_PRV** |  |  |  |  |
| Upper | 0 | 103 | 350 |  |

*gEUD: generalized equivalent uniform dose

**Table S4.** Geometric metrics of AI-generated prostate contours on MRI for 5 patients in our study cohort, with corresponding results on CT for comparison.

|  | **DSC** | **sDSC** | **MDA (mm)** | **HD95 (mm)** | **APL (pixels)** |
| --- | --- | --- | --- | --- | --- |
| **MRI** | | | | | |
| Patient 1 | 0.73 | 0.31 | 3.96 | 8.90 | 2168 |
| Patient 2 | 0.83 | 0.62 | 2.27 | 6.31 | 876 |
| Patient 3 | 0.14 | 0.00 | 12.43 | 19.63 | 2464 |
| Patient 4 | 0.68 | 0.32 | 4.17 | 8.06 | 1284 |
| Patient 5 | 0.59 | 0.27 | 4.88 | 12.53 | 1327 |
| mean ± (std) | 0.59 (±0.27) | 0.30 (±0.22) | 5.54 (±3.97) | 11.09 (±5.29) | 1624 (±664) |
| **CT** | | | | | |
| Patient 1 | 0.86 | 0.48 | 2.29 | 5.06 | 1464 |
| Patient 2 | 0.78 | 0.33 | 3.26 | 6.96 | 1335 |
| Patient 3 | 0.83 | 0.53 | 2.33 | 5.26 | 1234 |
| Patient 4 | 0.87 | 0.76 | 1.95 | 5.46 | 377 |
| Patient 5 | 0.63 | 0.26 | 5.15 | 15.01 | 1161 |
| mean ± (std) | 0.79 (±0.10) | 0.47 (±0.20) | 3.00 (±1.30) | 7.55 (±4.24) | 1114 (±428) |


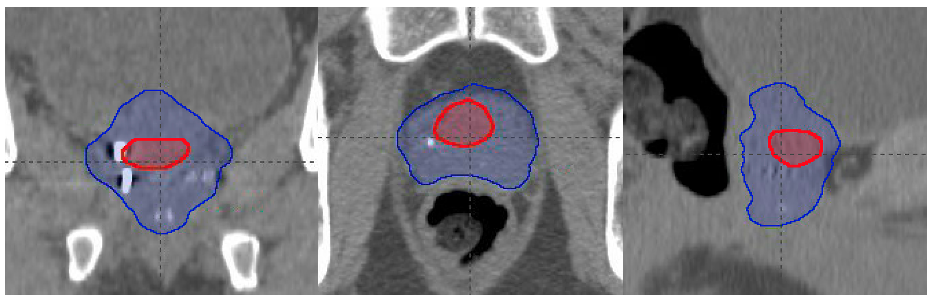
**Figure S1.** Example case (Patient 3 in Table S4) of AI-generated prostate contour on MRI (red) compared to the physician-delineated clinical prostate contour (blue) in three views.

***Ad hoc correlation analysis between geometric and dosimetric performance***

An *ad hoc* analysis was performed to investigate the correlations between geometric metrics and dosimetric endpoints using Pearson correlation (p < 0.05 considered significant), to explore the potential predictive value of the geometric metrics in dosimetric outcomes.

Significant correlations were observed in the rectum between geometric metrics, sDSC and APL, and dosimetric endpoints, as illustrated in Figure S2 and Figure S3, with corresponding Pearson correlation coefficients (r) and p-values provided. Stronger correlations were found with the post-processed AI rectum contours. As the rectum geometric metrics deteriorated (reflected by a decrease in sDSC and an increase in APL), its dosimetric endpoints tended to increase. No significant correlation was found between bladder geometric metrics and dosimetric parameters.


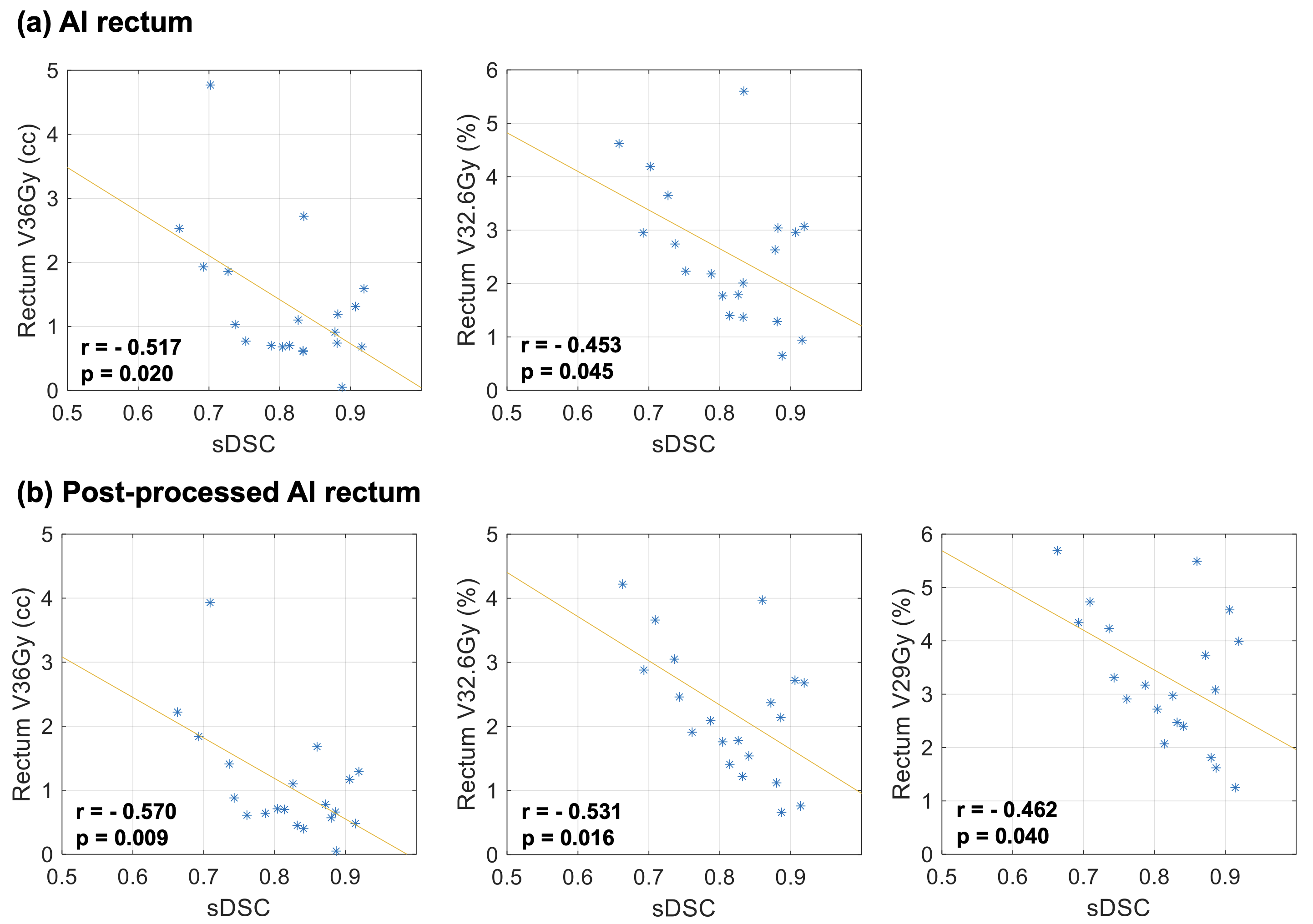


**Figure S2.** Significant correlations observed between rectum sDSC and dosimetric parameters V36Gy, V32.6Gy and V29Gy, with the Pearson correlation coefficients (r) and p-values (p) provided: (a) using AI rectum contours, (b) using post-processed AI rectum contours.


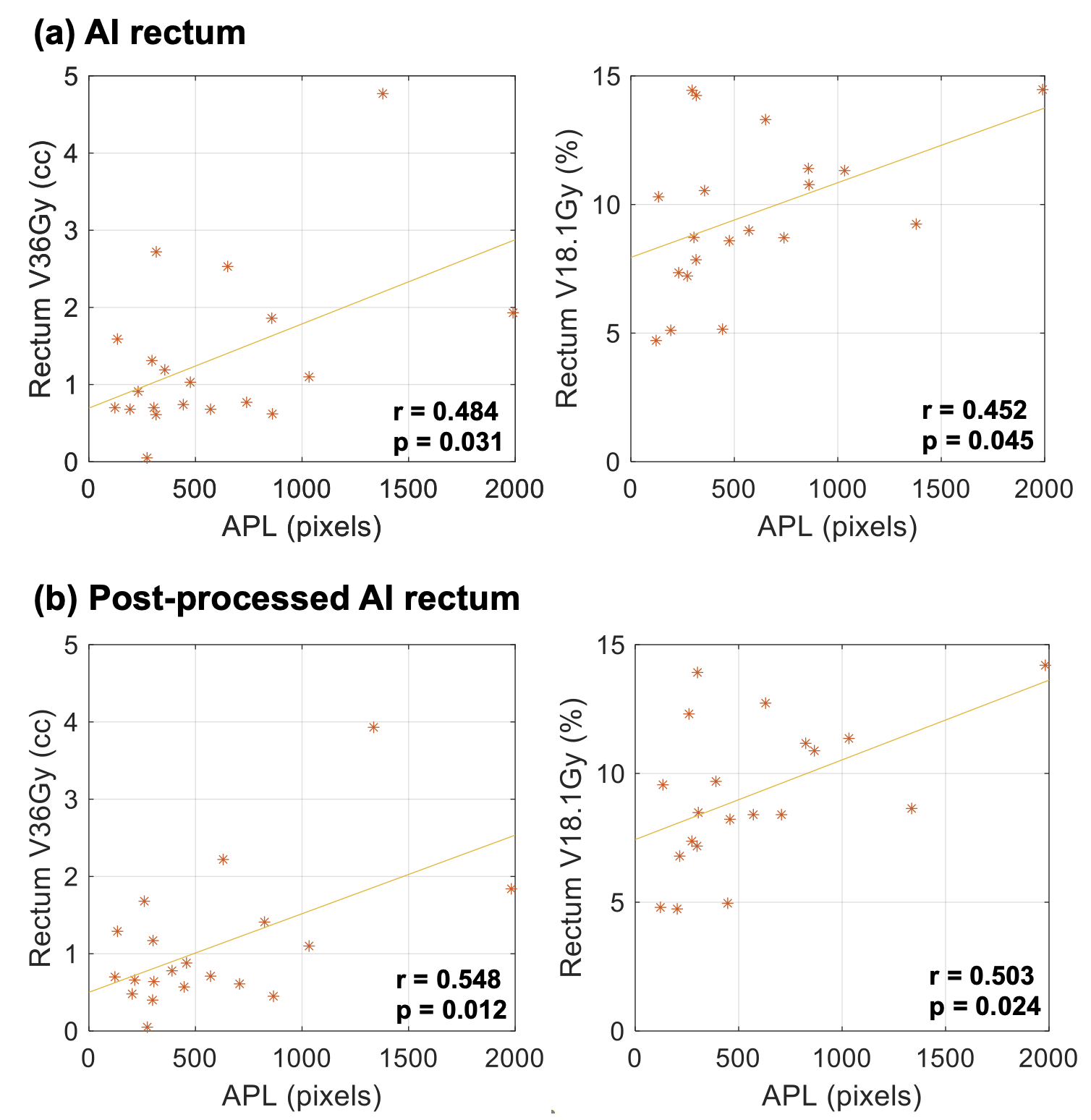


**Figure S3.** Significant correlations observed between rectum APL and dosimetric parameters V36Gy and V18.1Gy, with the Pearson correlation coefficients (r) and p-values (p) provided: (a) using AI rectum contours, (b) using post-processed AI rectum contours.
